# The Use of a Meckel's Diverticulum in the Creation of an Orthotopic Neobladder in Case of a Short MesoIleum: A Case Report

**DOI:** 10.1155/2009/493236

**Published:** 2009-10-20

**Authors:** Wim Van Haute, Christophe Ghysel, Peter Van Oyen

**Affiliations:** Urology Department, AZ St Jan Brugge, 8000 Brugge, Belgium

## Abstract

A 72-year-old patient was treated in our department for an invasive bladder TCC by cystoprostatectomy with the intention to create an orthotopic neobladder. During surgery it appeared to be impossible to mobilize part of the preterminal ileum into the small pelvis to make an anastomosis with the urethral stump. However, incidentally, a Meckel's diverticulum of about 8 cm was found on the preterminal ileum which could easily be mobilized onto the urethral stump. The intestinal insertion of the diverticulum served as the lowest point of the pouch. Above the diverticulum, we created a modified Studer-pouch. No major postoperative complications occurred and during the follow-up period of more than 12 months micturition was good.

## 1. Case Description

A 72-year-old patient was diagnosed with an invasive bladder carcinoma, with negative staging (PET-CT and bone-scan). Therefore, we planned to perform a radical cystoprostatectomy with the intention to form an orthotopic neobladder using 60 centimetres of preterminal ileum (modification of Studer-pouch). After an uneventful cystoprostatectomy, the terminal ileum could not be sufficiently mobilized to reach the urethral stump due to a short mesoileum.

**Figure 1:**
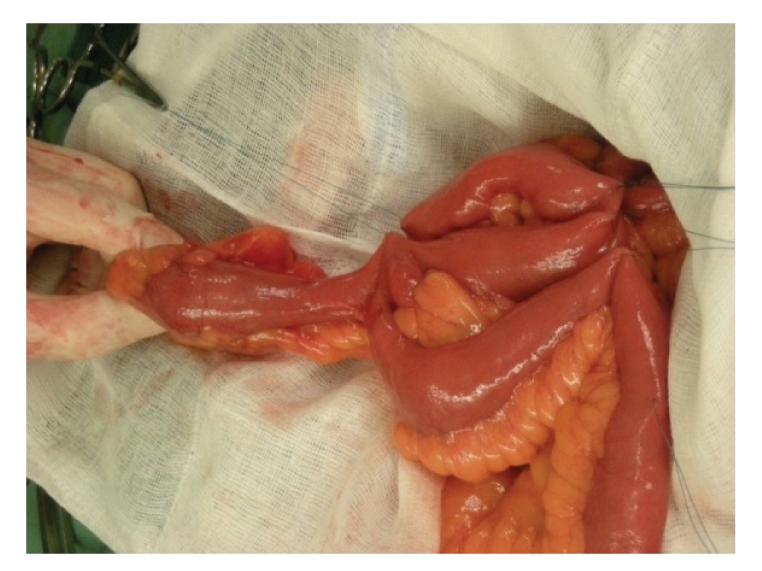
Meckel's diverticulum.

However, it so happened that a Meckel's diverticulum was found approximately 20 centimetres from the ileocaecal valve (Figure 1). The diverticulum was about 8 cm long, and the tip could be easily mobilized into the small pelvis where it reached the urethral stump.

**Figure 2:**
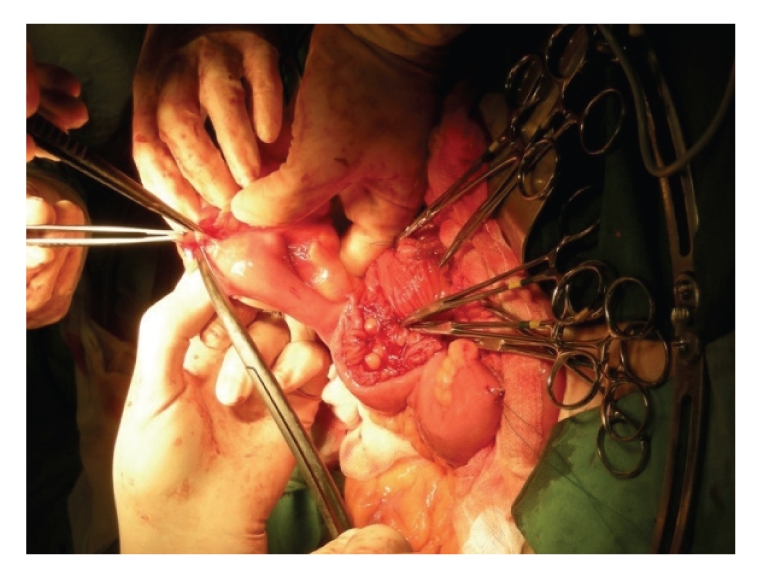
Creation of the pouch with Meckel.

The diverticulum was used to lengthen the neobladder onto the urethral stump. Above the diverticulum, a W-shaped pouch was created, with an afferent loop to the left on which the ureters were implanted (Figure 2).

Apart from a urinary infection which was treated with peroral antibiotics, no major postoperative complications occurred.

After removal of the transurethral catheter, the patient developed good micturition.

After one year of follow-up the neobladder functioned well. There was normal micturition without residual volume in the neobladder. After four months, daytime continence (1 pad or less/day) was fully recovered. After six months the level of nocturnal incontinence was again acceptable since the patient only needed one pad during the night.

## 2. Discussion

To our knowledge, this is the first report on the use of a Meckel's diverticulum in the creation of an orthotopic neobladder. There is a report, however, about the use of a Meckel's diverticulum to implant ureters to a Bricker derivation [1]. Also in nononcological urologic surgery, The Meckel's diverticulum has been used for ureteric repair [2] and as an alternative conduit for the Mitrofanoff procedure [3]. These are all case reports, except for Adams et al. [2], where the authors describe four cases.

We used the Pubmed search engine to screen all literature on the subject written between 1995 and present using terms as “neobladder”, “Meckel's diverticulum”, “urinary diversion” and “outcome”. We also performed a textbook study on urinary diversions.

A Meckel's diverticulum is present in 2 to 4% of the population, and it is the most common congenital malformation of the gastrointestinal tract [4]. It is understood to be an embryologic rest of the omphalomesenteric duct, which normally obliterates in the seventh week of gestation [5]. However, when the intestinal end of the duct persists, a Meckel's diverticulum is formed.

In most cases the mucosa of the diverticulum is that of the adjacent ileum, but it can also be gastric, duodenal or colonic [4]. The embryogenesis of these mucosal tissues is unknown [4]. A Meckel's diverticulum is normally diagnosed due to a complication, such as a gastro-intestinal bleeding in 25 to 50% of the cases, or an infection in about 20% of the cases [4]. There is less risk of complications in patients over 40–50, when there is no ectopic mucosa and smaller length of the diverticulum. Complications are mostly treated by performing a diverticulectomy.

The objective of the techniques described for an orthotopic neobladder is to create a low-pressure reservoir which is capacious and continent [6]. The different techniques described all seem to have a similar outcome and morbidity. If about 40 cm of bowel is used, this should not lead to any malabsorption nor to a major bowel dysfunction. In most patients, the ileum can easily be manipulated into the pelvis without tension. Furthermore several studies have shown that perioperative morbidity is acceptable, even in older patients [7]. Therefore, the ileal orthotopic neobladder is considered to be an excellent choice for a continent urinary diversion after radical cystectomy [6]. In some cases, however, the mesoileum is too short, and no part of the ileum can be mobilized enough to make an anastomosis on the urethral stump. The surgeon normally has to use a Bricker-type ileal conduit and create an external stoma.

This would also be the case for the patient described. However, the Meckel's diverticulum we found was in a very fortunate location, and the tip could be easily mobilized into the small pelvis.

We used the full length of the Meckel's diverticulum to achieve an anastomosis with the urethral stump without traction. The intestinal insertion of the diverticulum was considered to be the lowest point of the pouch. A W-shaped pouch was created above the diverticulum. The ureters were implanted on a short chimney. This operative technique enabled us to create a good pouch and to reach the urethra without traction.

In our opinion, the use of the diverticulum is safe because of the patient's advanced age, the absence of any inflammatory bowel disease [4], and the appearance of a normal ileal mucosa on inspection. Another reason to persist in the creation of this type of neobladder was the fear that this particular patient had of an ileal conduit with a stoma.

However, several studies, such as Autorino et al. [8], show that the postoperative Quality of Life (QoL) after an orthotopic continent bladder substitution, and an ileal conduit with a stoma are very similar. They advise to sufficiently educate and inform the patient about the different options since this is most likely to lead to postoperative satisfaction.

## 3. Conclusion

We described the successful use of a Meckel's diverticulum to lengthen the distal part of an orthotopic neobladder in a patient with an impossibility to move the preterminal ileum into the small pelvis.

Because of the extreme rarity of the concomitant appearance of a Meckel's diverticulum and a short meso-ileum in a patient needing a cystectomy with a continent diversion, no description of a similar case can be found in literature. However, it seems that in the rare case of these three factors coexisting, this approach can be considered. The patient's advanced age and the normal appearance of the diverticulum is contraindicative for diverticular complications. Postoperative recovery of the micturition and continence function were also satisfying.
